# The histone chaperoning pathway: from ribosome to nucleosome

**DOI:** 10.1042/EBC20180055

**Published:** 2019-03-22

**Authors:** Alonso J. Pardal, Filipe Fernandes-Duarte, Andrew J. Bowman

**Affiliations:** Division of Biomedical Sciences, Warwick Medical School, Gibbet Hill Road, Coventry CV4 7AL, U.K.

**Keywords:** Chaperone, chromatin, HIstone chaperone, histones, Nucleosome

## Abstract

Nucleosomes represent the fundamental repeating unit of eukaryotic DNA, and comprise eight core histones around which DNA is wrapped in nearly two superhelical turns. Histones do not have the intrinsic ability to form nucleosomes; rather, they require an extensive repertoire of interacting proteins collectively known as ‘histone chaperones’. At a fundamental level, it is believed that histone chaperones guide the assembly of nucleosomes through preventing non-productive charge-based aggregates between the basic histones and acidic cellular components. At a broader level, histone chaperones influence almost all aspects of chromatin biology, regulating histone supply and demand, governing histone variant deposition, maintaining functional chromatin domains and being co-factors for histone post-translational modifications, to name a few. In this essay we review recent structural insights into histone-chaperone interactions, explore evidence for the existence of a histone chaperoning ‘pathway’ and reconcile how such histone-chaperone interactions may function thermodynamically to assemble nucleosomes and maintain chromatin homeostasis.

## Introduction

**Figure 1:**
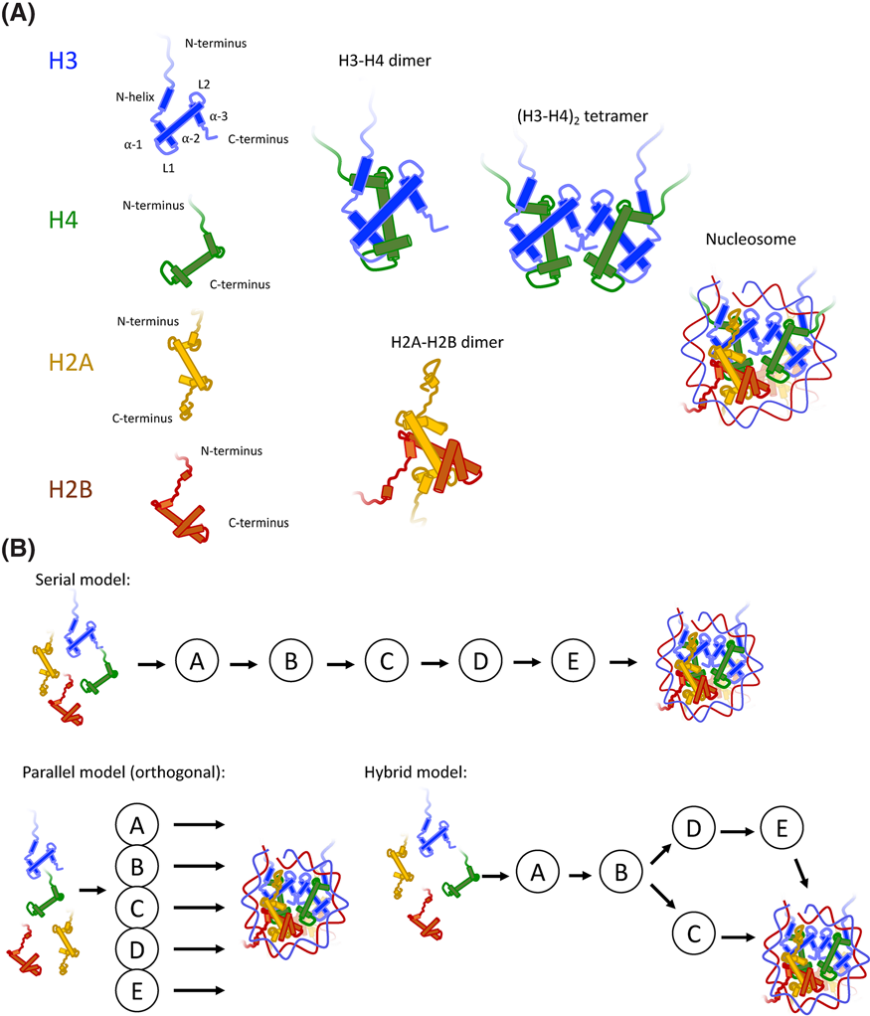
Components of the nucleosome and considerations for multichaperone networks (**A**) Nucleosomes comprise of four core histones – H2A, H2B, H3 and H4. The central histone fold comprises three helices, α1, α2 and α3, which can be flanked by extension on the N and C termini. N-terminal extensions are in the form of basic tails, and in the case of H3, an additional α-helix (αN-helix). H3 and H4 fold to form an H3–H4 dimer, two of which can form an (H3–H4)_2_-heterotetramer. The tetramer adopts the central location in the nucleosome, known as a ‘tetrasome’ when bound to DNA on its own, and is capped by two H2A–H2B dimers to form the nucleosome core particle, which wraps 145–147 base pairs of DNA in almost two superhelical turns. (**B**) Histones associate with a large repertoire of chaperoning complexes, represented as A, B, C, D and E. Assuming mutual exclusivity in binding, histones may transition through multiple chaperoning complexes before their incorporation in chromatin (a serial pathway), or each chaperoning complex may represent an orthologous route to deposition (a parallel pathway), or indeed, a mixture of the two (a hybrid model). In reality, a hybrid model which bifurcates depending of histone isotype best explains the H3–H4 deposition pathway (see Figure 2).

Eukaryotes package their genome within the confines of the cell nucleus into a structure broadly defined as chromatin. Chromatin is composed of an array of proteins, RNA and genomic DNA, whose integrity is crucial for genomic regulation, stability, replication and repair [1,2]. The most abundant proteins in chromatin are histones. These form an octamer around which genomic DNA wraps. Two copies of Histone H2A, H2B, H3 and H4 are encircled by a strand of DNA that is wrapped 1.65 times, corresponding to ∼147 base pairs of DNA [3]. These core histones, together with their associated DNA form the basic repeating unit of chromatin – the nucleosome (Figure 1A). Core histones contain a globular region that is characterized by a conserved histone-fold domain. Histone-fold domains comprise approximately 65 amino acids that form three α-helices in the presence of a dimerization partner [4]. On the N-terminus, histones present an unstructured tail that protrudes from the nucleosome when fully assembled, and can also contain C-terminal extensions [5]. Dimerization of histones occurs by the antiparallel association of histone monomers in what is often referred to as a ‘handshake’ motif [6] (Figure 1A).

Histones are positively charged at physiological pH, enabling them to interact with the negatively charged DNA backbone. However, this also means histones are prone to promiscuous interactions with acidic cellular components, which may result in spurious protein aggregates. As such, regulating histone interactions from their synthesis in the cytoplasm and subsequent integration into nucleosomes is paramount. This function is performed by proteins that interact with soluble (i.e. non-chromatinized) histones, broadly defined as ‘histone chaperones’.

Histone chaperones originate from a wide range of protein families, spanning diverse protein folds [7]. Yet, they share the key common feature of binding histones and protecting them from undesirable interactions, be that during storage, transport, or nucleosome assembly [8]. Here we present an overview regarding the life of a histone, from synthesis and ribosomal exit, through import and nuclear processing, and finally deposition onto DNA at sites of DNA replication or histone turnover.

## The challenges in observing transitions between histone chaperones

Core histones stably interact with a large repertoire of structurally distinct histone chaperones [9]. As histones are small proteins (a histone fold dimer is approximately 25 kDa) it is unlikely that all of these proteins can bind at once, and indeed, more detailed biochemical investigations have demonstrated, for H3 and H4 at least, both compatibility and mutual exclusivity in histone binding [10–15]. These findings as a whole have been corroborated through high-resolution structures demonstrating both competition and compatibility. The question thus arises of whether mutual exclusivity in histone chaperone binding manifests as multiple parallel pathways for histone deposition, or whether a serial pathway prevails, in which histones transition between chaperoning components, or whether a mixture of the two exists in the cell (Figure 1B).

**Figure 2:**
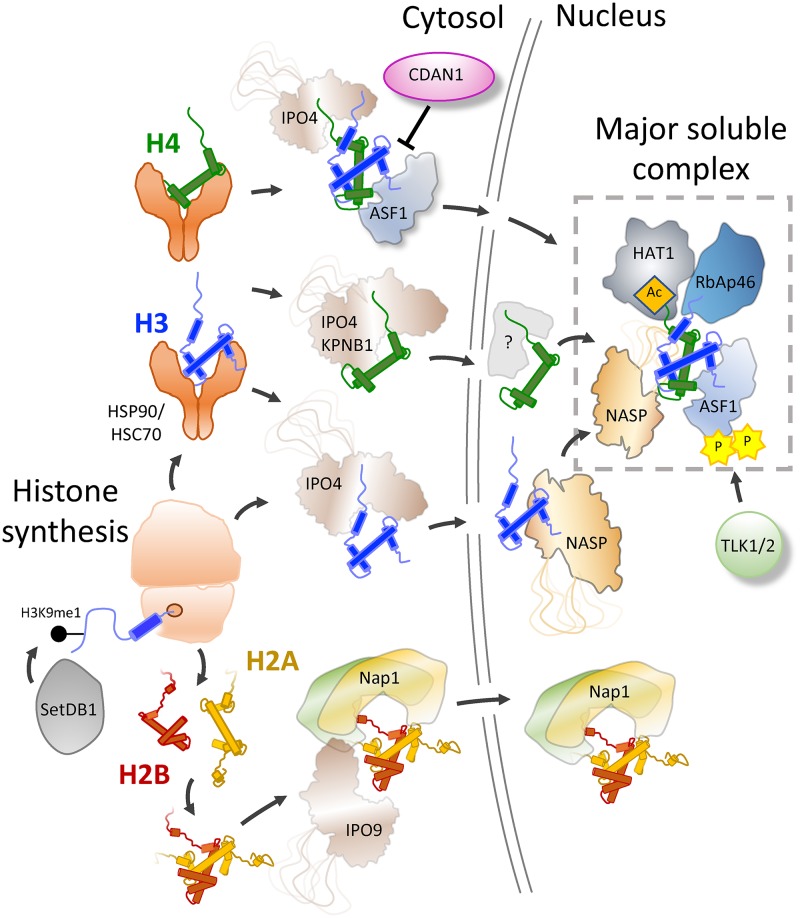
Histone synthesis and nuclear import After synthesis histones H3 and H4 interact in the cytosol with common folding chaperones HSC70 and HSP90, with H3 being mono-methylated on K9 co-translationally by SetDB1. Two pathways have been proposed for the import of H3 and H4: folding of an H3–H4 dimer in the cytosol and subsequent binding to ASF1 before import by IPO4, or import of monomeric histones bound directly to importins, with H3–H4 dimerisation occurring in the nucleus. ASF1’s interaction with H3–H4 in the cytosol can be regulated by the protein CDAN1, which competes with histone for ASF1 binding. H2A and H2B are imported as dimers bound to NAP1 and IPO9. In the nucleus, NASP interacts with H3 downstream of ASF1 through a high affinity interaction with the H3 α3 helix, and may thereby function as a receptor of H3 from the import machinery. Whether a monomeric H4 chaperone exists is not known. RbAp46 and HAT1 associate with H4, acetylating K5 and K12, and together with NASP and ASF1 form the major soluble H3–H4 complex. The S-phase-specific TLK1 and TLK2 can increase the affinity between histones and ASF1 through ASF1-specific phosphorylation marks.

**Figure 3:**
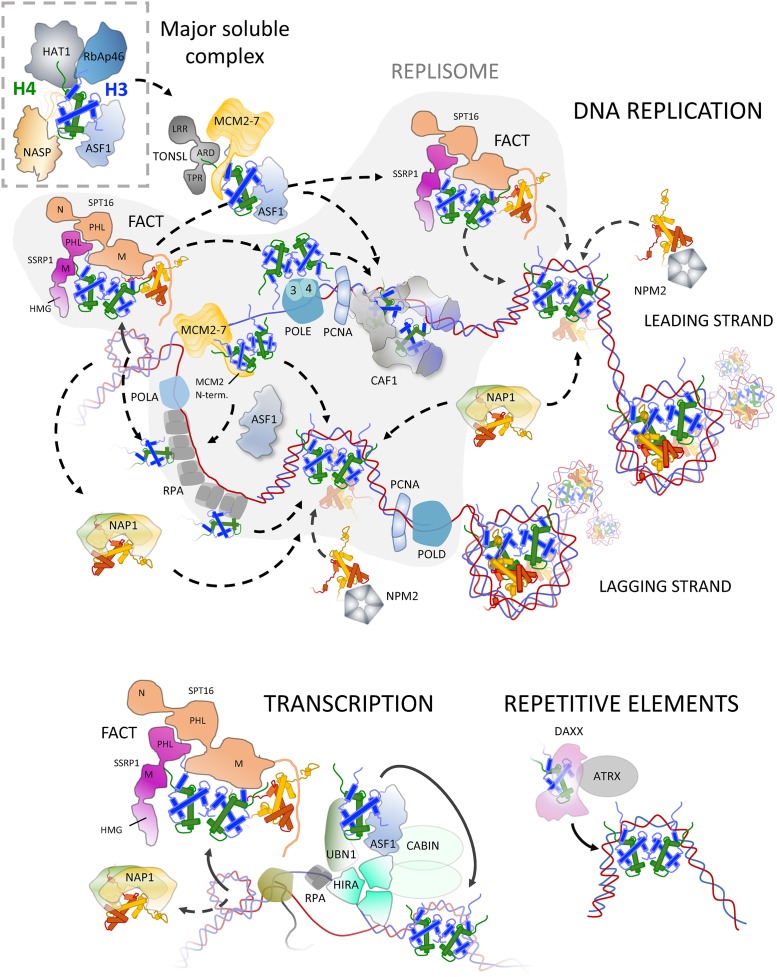
Histone chaperoning in the nucleus Histone deposition can be split into replication dependent, and replication independent mechanisms. The variants H3.1/H3.2–H4 are incorporated into chromatin predominantly at the replication fork during replication dependent deposition, in which a number of chaperoning components have been implicated, including FACT (SpT16-SSRP1), CAF1 (p150, p60, RbAp48), and the replication proteins POLE3–4, RPA, MCM2 (N-terminal tail) and TONSL. TONSL is also involved in the DNA damage response, a role that may be related to its chromatin assembly function. ASF1 may act as a bridging factor between soluble pool and deposition factors. In addition to H3–H4, the FACT complex also binds to H2A–H2B through its U-turn motif and C-terminal acidic stretch, and may be responsible for governing H2A–H2B deposition post replication, or indeed, the chaperones NAP1 and Nucleoplasmin may play a role. Replication independent deposition occurs throughout the cell cycle, utilising the H3.3 variant. At sites of transcription both the HIRA complex (HIRA, UBN1 and CABIN1), which can bind naked DNA, and FACT complex, which associates with elongating RNA pol II, are involved in H3–H4 deposition, with the FACT complex also extending its role to H2A–H2B incorporation. At other sites in the genome, including heterochromatic repetitive elements, the chaperone DAXX and chromatin remodeller ATRX govern the incorporation of H3.3. Histone chaperones also play a central role during DNA repair, omitted here for brevity. For a detailed description we refer the reader to some recent excellent reviews [131–133].

Experimentally, gaining kinetic information on the protein-protein interactions that histones make from the point of synthesis to the point of deposition has been difficult. Early pulse-chase analysis using radiolabelled amino acids and cultured mammalian cells revealed that newly synthesised histones are incorporated into chromatin in less than a minute after their synthesis [16]. The challenging nature of performing a pulse-chase experiment, that also relays protein-protein interaction information on such short time scales means the majority of our understanding has been derived through structural and biochemical observations, often of individual components in isolation. Taking histones H3 and H4 as examples, this has resulted in a number of common themes: (1) Histones are imported through interaction with importin-β proteins, namely IPO4, (2) a multi-chaperoning complex involving ASF1a/b, NASP, HAT1 and RbAp46 forms the major soluble pool of H3 and H4, (3) ASF1 is a central hub for the histone chaperoning network and is regulated by a number of co-chaperoning factors including the Tousled-like kinases 1 and 2 (TLK1 and TLK2), and Codanin-1 (CDAN1) in humans, and (4) H3–H4 are transferred to variant specific chaperones, often tethered to chromatin, for incorporation at sites of DNA replication or histone turnover (Figures 2 and 3). Thus, it appears that histones H3 and H4 pass through a common soluble complex, regardless of their variant type (excluding the centromeric variant CENPA), before they bifurcate depending on isoform and/or genomic context into a number of different deposition complexes. Much less is known about potential hand-off between H2A–H2B chaperoning complexes, therefore, in this article we will focus predominantly on the chaperoning pathways concerned with H3 and H4.

## Histone synthesis and nuclear import

Shortly after translation, histones interact with canonical folding chaperones. H4 has been shown to interact with HSC70 and HSP90 [11], whereas H3 interacts solely with HSC70 [17]. Attempts have been made to separate out pre- and post-import machineries through biochemical fractionation of the cytosolic fraction. However, a number of factors identified to be cytosolic in these studies have been found to be nuclear when probed in intact cells; namely NASP [15,18], ASF1 [19–21], HAT1 [22,23] and RbAp46 [15,24,25]. The reason for this discrepancy could lie in the soluble nature of these proteins, rapidly leaking into the cytosolic fraction upon common sub-cellular fractionation methods, with insoluble proteins, such as lamins and tubulin, being poor controls for reporting on such mixings [15,26].

Biochemical data showing a soluble pool of importin-ASF1 bound to the H3–H4 dimer suggested a fast mechanism for histone H3 and H4 co-folding and import [10,11,17]. Recently, evidence combining cell microscopy and a novel pulse-chase system to deploy fluorophore-labelled histones, suggested that human histones H3 and H4 can be translocated by importin proteins as monomers to the nucleus [15]. It should be highlighted, however, that the approach required a synthetic tethering system for observation, and therefore its relevance to endogenous histone import had to be implied. Promisingly, however, it was also found that H3 exists in a stoichiometric excess of H4 when bound to the ubiquitous nuclear chaperone NASP, suggesting an endogenous pool of NASP-H3 monomer may exist (Figure 2) [15]. This was in support of previous biochemical analysis of NASP showing that it could form both a tetrameric complex with H3–H4 and ASF1, and a stable dimeric complex with H3 on its own, albeit using purified components *in vitro* [27]. Further investigation is needed to discriminate the utilisation of these two potential pathways for histone import.

NASP is the human homolog of the first H3–H4-specific chaperone to be identified, N1/N2 [28,29], and contains four conserved TPR repeat motifs. Stacking of TPR repeats forms a superhelical groove that often serves as a peptide binding pocket in mediating protein-protein interactions [30]. Consistently, it was found that NASP binds to a short motif found at the very C-terminus of histone H3 through a canonical TPR-peptide interaction [31], but with much greater affinity than is typical of TPR–peptide interactions. Interestingly, the crystal structure of ASF1–H3–H4 shows that the residues of H3 involved in NASP interaction are the same residues necessary for binding to ASF1 [32–34]. NASP and ASF1 can compete for this binding site, with NASP out-competing ASF1 when an H3 monomer is used as a substrate [27]. Surprisingly, however, NASP can also bind an H3–H4 dimer in a conformation that is compatible with ASF1 [27]. This disparity was resolved with the discovery of a second H3–H4 specific interaction site on NASP involving its unstructured acidic domain [27]. Interestingly, the ability to chaperone both H3 monomers and H3–H4 dimers implies that NASP may play a role in the folding of H3 with H4. Its ability, in concert with ASF1, to produce folded H3–H4 dimers *in vitro* from monomeric substrates lends support to this idea [27]. Governing the transition from monomer to dimer may also aid in NASP’s ability to protect a soluble pool of H3–H4 *in vivo* [35]. Thus, NASP may function as a receptor for incoming monomeric H3 in the nucleus, accepting H3 from importins, and guiding its folding with H4. In addition to NASP, the histone chaperone ASF1 has the ability to destabilize the importin-histone H3 interaction *in vitro* [36], suggesting a possible role for importin-histone dissociation upon nuclear localization.

HAT1 and RbAp46 form the dimeric HAT1 complex, which can interact with H3–H4 together with ASF1 and NASP [13–15,37]. This is likely due to the binding sites of HAT1 and RbAp46 residing in the N-terminal tail [38] and first α-helix of H4 [23,39,40], respectively, being distinct from the binding sites of NASP and ASF1. A recent co-crystal structure of the yeast HAT1 complex demonstrated how binding of the α1 helix by HAT2 (the yeast homolog of RbAp46) positions the H4 tail for acetylation on K12 by the enzyme HAT1 [41]. Interestingly, the binding site of HAT2 (RbAp46), the α1 helix of H4, is positioned against the H3–H4 histone fold dimer in all known crystal structures. Thus, in order to interact with RbAp46 the helix must rotate outwards, suggesting that the H3–H4 dimer may be in a destabilised form when associated with the HAT1 complex.

Diacetylation of newly synthesised H4 is highly conserved throughout evolution, however, its mechanistic function is still unclear, with the modification being removed soon after deposition. In addition to diacetylation of H4 [42], recent biochemical analysis of H3 has proposed methylation of K9 by SETD1B in the cytosol, occurring co-translationally [43]. Such methylation on newly synthesised H3 has been proposed to potentiate their final epigenetic fate [44–46]. Similarly, the enzymes PRDM3 and PRDM16 have been shown to mono-methylate H3K9 in the cytosol of mouse cells, a necessary precursor of tri-methylation in the nucleus [47]. HAT2 was shown to interact with the N-terminus of H3 through interacting with unmethylated R2 via the pocket formed at the centre of its WD40 repeats [41]. As newly synthesised H3 molecules are devoid of R2 methylation, it was suggested that this could be a mechanism to discriminate between new and old histones. In conclusion, the major soluble complex of H3–H4 in human cells is non-variant specific in relation to H3, and contains the chaperoning components ASF1, NASP, RbAp46 and HAT1 (Figure 2).

## Incorporation of newly synthesized histones

Numerous H3–H4 specific chaperones, in addition to established replication components with novel chaperoning ability, are stable constituents of the replication fork, including CAF1, FACT, MCM2, ASF1, RPA, POLE3–4 and TONSL [48–56]. During replication-dependent deposition both parental and newly synthesised histones must be used as substrates to assemble the newly replicated DNA into chromatin.

H3–H4 dimers can be provided by *de novo* nucleosome assembly through ASF1. Interestingly, ASF1, a component of the major soluble complex of H3–H4 (Figure 3), can interact with a number of deposition chaperones including CAF1, MCM2 and HIRA. This suggests a role for ASF1 in bridging the soluble and deposition phases of the histone chaperoning pathway. ASF1 in humans is present in two non-allelic isoforms, ASF1a and ASF1b. These paralogs share 70% sequence identity, and appear to be predominantly redundant in their roles as histone chaperones, although ASF1b is associated with cellular proliferation, whereas ASF1a is also expressed in cells that have become quiescent [57].

ASF1 can interact with an H3–H4 dimer at the same time as the unstructured N-terminal region of MCM2 and the ankyrin repeat domain (ARD) of TONSL [52,58]. In turn, MCM2 has been shown to bind to both the (H3–H4)_2_ histone tetramer and to ASF1 bound to an H3–H4 dimer [59,60], the latter suggesting a mechanism for the recycling of (H3–H4)_2_ histone tetramers across the replication fork [59,61]. TONSL interacts with an H4 tail peptide through its ARD domain, which can occur concomitantly with MCM and ASF1 binding [58]. Interestingly, the MMS22L–TONSL complex remains bound to H4 after its incorporation into chromatin, marking post-replicative chromatin through its ability to discriminate newly synthesized, non-methylated H4K20 [58]. TONSL has an additional, well documented, role in stimulating replication fork recovery after collapse, a function that may be linked to its chromatin assembly ability [52,53]. As TONSL interacts selectively with newly synthesised H4, and is found in complex with MCM2, it follows that MCM2 may also interact with newly synthesized histones in addition to parental histones.

ASF1 also interacts with yeast CAF1 subunit Cac2 [62–65] and its human [21] and fly counterparts [19] through a weak interaction distinct from its H3–H4 binding interface [66,67], providing the H3–H4 dimer for the concerted H3–H4 tetramerization on DNA by a CAF1 dimer. The small subunit of CAF1, RbAp48, interacts with the α1 helix of H4 [23] and likely represents a secondary hand-off event from its paralog RbAp46 in the HAT1 complex [39,68]. The CAF1 complex is recruited to active replication foci [69], where it binds to the DNA polymerase complex through its PCNA interacting peptide (PIPs) motifs [49,70–73]. *In vivo* [74] and *in vitro* [75] studies have shown that CAF1 is necessary and sufficient for nucleosome deposition after DNA replication. In yeast, Cac1, the largest subunit, acts as a scaffolding for the Cac2 and Cac3 subunits as well as for the H3–H4 dimer [76]. The H3–H4 dimer binds extensively to the Cac1’s C-terminus, including an acidic domain that releases intramolecular interactions with the Winged-helix DNA Binding domain, or WDB [77]. In the proposed model, the freed WDB domain can then bind to DNA, stabilising the complex at the replication fork [78]. The Cac1 subunit undertakes most of the chaperoning activity, enabling CAF1 complex dimerization, and promoting the deposition of H3–H4 onto DNA as a tetramer (H3–H4)_2_ [76,77].

## Histone recycling during replication

Early experiments established the ‘tetramer conservation model’, where (H3–H4)_2_ tetramers are formed either with parental H3–H4 or *de novo* synthesized histones, but not randomly mixed [79,80]. Parental and new tetramers are found evenly partitioned across the leading and lagging strands, forming full nucleosomes in a second step with available H2A–H2B dimers [80–82]. However, the tetramer conservation model is not completely universal, but rather restricted to replication, since highly translated genes tend to present a certain degree of tetramer mixing [83], contrary to lowly expressed loci [84]. In agreement with this, tetramers with histone variant H3.3, associated with the replication-independent pathway, are more likely to mix than tetramers with variant H3.1 deposited during DNA replication [85].

Nucleosome recycling constitutes a potential mechanism to enable the perpetuation of the chromatin landscape with its implications in epigenetic inheritance. Experiments recreating replication *in vitro* suggest that parental nucleosomes tend to remain at nearby loci when a rich extract containing all the replication-fork interacting chaperones (such as the aforementioned FACT, CAF1, or MCM2) is used [86]. On top of this, nascent chromatin sequencing paired with amino acid radio-labelling suggests that PTM are kept on parental histones with chromatin landscape reconstruction within one generation [87]. Due to the speed with which replication occurs, it has been difficult to gain direct information about the order of binding events that must follow to process both newly synthesised and parental histones, and the mechanisms that may keep them partitioned. However, significant advances have been made from recent structural, biochemical and novel next-generation sequencing.

Replication protein A complex (RPA, a complex that binds and protects single strand DNA), can also function as a binding partner for an H3–H4 dimer [55]. Possibly related to these findings, recent studies have speculated that histones may stably interact with single-stranded DNA [88,89]. Whether this is mediated through their interaction with RPA, and what role this would have *in vivo* (perhaps protecting the lagging strand or conserving the original nucleosome position), is yet to be solidified. Interestingly, DNA polymerase ε (POLE) small subunits POLE3 and POLE4 have recently been reported to act as H3–H4 histone chaperones at the replication fork during histone recycling [56] (Figure 3). Work done on yeast homologs Dpb3 and Dpb4 suggests that these subunits of DNA polε favour parental histone loading on the leading strand to counter a lagging strand bias [90]. Conversely, MCM2 has been implicated in the even distribution of parental (H3–H4)_2_ tetramers across both DNA strands, showing a preference for parental histone recycling to the lagging strand in mice to counter a leading strand bias [91,92]. Future work is necessary to clarify *in vivo* deposition partners and their handover mechanisms. For instance, do POLD3 and POLD4 (equivalent subunits to POLE3 and 4 in polymerase δ) have similar roles in the lagging strand?

## Nucleosome disassembly, re-assembly and H2A–H2B histone chaperones

Another highly conserved chaperoning complex at the replication fork is FACT. FACT comprises three subunits in yeast, Spt16, Pob3 and Nhp6, but only two, SPT16 and SSRP1, in metazoans [93]. Originally identified in its role as FACilitating Transcription (FACT) through its interaction with RNA polymerase II [94–96], FACT has an essential role in the removal [97] and deposition of nucleosomes [98], and localizes to the replication fork predominantly through interactions with DNA polymerase α [50,99], but also through MCM2 [54] and RPA [100]. FACT can bind to H2A–H2B dimers through both the U-turn motif of the Spt16 M-domain, and through hydrophobic residues within the C-terminal acidic domains of both Spt16 and Pob3 [99,101]. In addition, the Spt16 subunit can bind to the (H3–H4)_2_ tetramer through the M-domain, contacting the same surface used to mediate histone–DNA interactions [102]. SSRP1 can bind to both H3–H4 and H2A–H2B [103]. FACT binds simultaneously to the (H3–H4)_2_ tetramer bound to DNA and to H2A–H2B [104], possibly interacting with the H2A–H2B dimer in concert with DNA polymerase α [105]. The structure of Spt16 bound to the (H3–H4)_2_ tetramer is in contrast with that of MCM2 in that it contacts both H3–H4 dimers across the dyad interface, rather than a single H3–H4 dimer. This may have implications in the re-deposition of the parental tetramer across cell generations as mentioned above [80,84,85].

Functionally, *in vitro* nucleosome reconstitution and electrophoretic mobility assays showed that FACT can bind transiently to partially dissociated nucleosomes [101,104]. Structural modelling suggested that FACT acts as a wedge binding to the (H3–H4)_2_-tetramer whilst still associated with DNA to favour H2A–H2B dissociation [102]. Furthermore, micrococcal nuclease treatment of FACT-bound nucleosomes and nucleosome fragments evidence that FACT binds preferentially to hexasomes or partially digested nucleosomal DNA rather than to whole nucleosomes [102,104]. These observations, together with the ability to reconstruct nucleosomes *in vitro*, where DNA ultimately outcompetes FACT [104], suggests that FACT is capable of disassembling nucleosomes only when the process is energetically favourable (potentially fuelled through the CMG helicase, a processive polymerase or an ATP-dependent chromatin remodelling factor).

In addition to FACT, NAP1 and Nucleoplasmin, as discussed in the next section, are H2A–H2B binding chaperones that could provide H2A–H2B for nucleosome assembly behind the replication fork. Although they have been isolated as constituent parts of the replisome, they could be in high enough concentration in the nucleus that free diffusion is sufficiently rapid to allow the final step of nucleosome assembly to occur within a sufficient time frame.

## Histone chaperoning during transcription

Outside of S-phase, histones are deposited at regions of high nucleosome turnover. This occurs as a consequence of processes requiring access to DNA, such as transcription, DNA repair, recombination and also variant replacement under specific genomic contexts [106]. HIRA is a histone chaperone complex, named after its principle subunit, dedicated to nucleosome formation in a replication-independent manner [13,107]. HIRA functions by chaperoning H3.3 variant to exposed DNA and transcriptionally active regions [108–110]. The HIRA complex is made of three HIRA subunits, two CABIN1 subunits and Ubinuclein-1 or UBN1 [111]. ASF1 serves as histone H3.3–H4 dimer donor through the B-domain of HIRA, analogous to that of the recruitment to CAF1 [112,113]. The specificity of binding towards H3.3 is conferred to the HIRA complex through the UBN1 subunit, directly interacting with ASF1–H3.3–H4 [114]. HIRA complex can be tethered to actively transcribed DNA through RPA [115], highlighting the universal nature of RPA tethering chromatin formation to single stranded DNA. Indeed, a gap-filling mechanism has been proposed for HIRA in which it senses naked DNA through its intrinsic DNA-binding ability and directs deposition of an H3.3 containing nucleosome [109].

In addition to being a component of the replisome, FACT is also an essential chaperone that associates with elongating RNA polymerase II, as its name suggests (FACilitating Transcription) [94,116], as well as with RNA polymerase I [117], and III [95]. One can assume that the underlying biochemical mechanism for nucleosome reorganisation by FACT is the same for both its functions at the replication fork and at sites of transcription, and again suggests that FACT is only capable of outcompeting DNA for its substrate when fuelled by a processive enzyme such as RNA polymerase. Kinetic analysis suggests that human FACT decreases the non-productive time of RNA polymerase II [118] while ChIP-seq analysis showed a marked correlation between FACT associated with gene transcription start sites and gene transcription [117]. Taken together, these data suggest a role for FACT in helping RNA polymerase II processivity and ensuring the prompt reassembly of the nucleosome (reviewed in [119]).

Structurally similar to FACT, ANP32E is a histone chaperone dedicated to the removal of histone variant H2A.Z–H2B dimers, located to actively transcribed genes and regulatory elements such as enhancers and insulators [120]. Another H2A–2B chaperone that has been implicated in transcription is nucleosome assembly protein 1 (Nap1). Nap1 envelops the H2A–H2B dimer similarly to DNA [121], preventing non-productive associations and thereby driving correct nucleosome assembly [122]. *∆Nap1* assays in yeast showed an increase in the presence of hexasomes in the genome, suggesting a preferential role in depositing the second of the two H2A–H2B dimers [121]. Nucleoplasmin (or NPM2 in humans) is a pentameric (or decameric) complex [123,124] that can reconstitute nucleosomes *in vitro* [125]. The potential interactions and dynamics between H2A–H2B-interacting chaperones, such as FACT, Nap1 and nucleoplasmin remain to be clarified.

## Histone replacement at repetitive elements

Interestingly, the H3.3 variant accumulates not only on actively transcribed genes, but also over largely silenced regions such as telomeres and pericentromeric regions [103]. This observation led to the discovery of the histone-binding protein DAXX and chromatin remodeler ATRX, that form a dimeric complex involved in H3.3 deposition and remodelling [23,29]. DAXX envelops the H3.3–H4 dimer in a way incompatible with ASF1 interaction, and correspondingly ASF1 and DAXX are not found in complex with each other *in vivo*. Whether H3.3–H4 must first pass through ASF1 and the major soluble H3–H4 complex, or whether DAXX represents an orthologous pathway to H3.3 deposition has yet to be determined. The specificity of DAXX to histone H3.3 over H3.1 relies on the presence of a glycine residue at position 90 of histone H3.3 (one of the five amino acids that vary between H3.1 and H3.3) in such a way that any substitution abrogates the complex formation [126]. ATRX recruits DAXX to regions enriched in heterochromatic marks such as H3K9me3 [127], the presence of heterochromatin protein 1 HP1 [128] or through the mediation of long non-coding RNAs [129]. In turn, ATRX/DAXX complex plays a role in maintaining and propagating this repressive chromatin state [130], working as a positive feedback loop.

## Conclusions and future outlook

**Figure 4:**
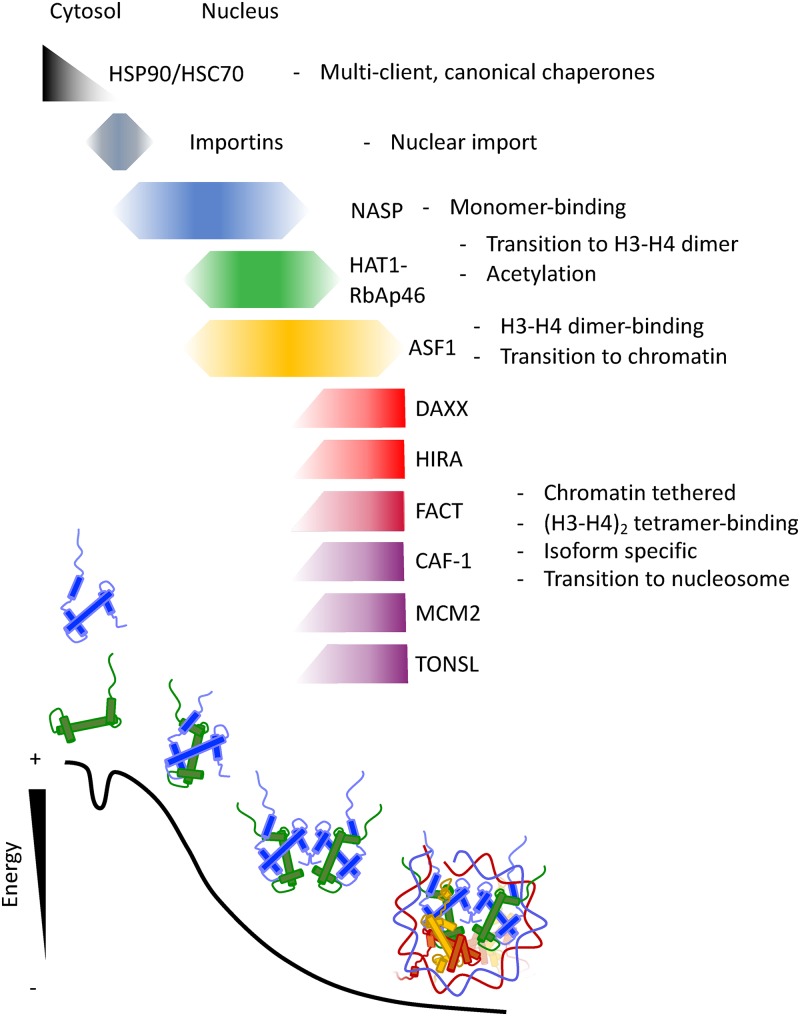
Transitioning through the thermodynamic landscape of nucleosome assembly Histones must transition through a number of protein complexes in order to fold with DNA into nucleosomes without the input of energy from ATP hydrolysis. Immediately after synthesis histones contain a high free energy. This energy may be captured by histone chaperones and utilised in a way which drives correct folding and oligomerisation of histone subunits. This is mostly likely achieved through extensive and specific interactions covering all transitions states of histone intermediates. In such a scenario, accepting potential ATP-driven input from canonical protein folding chaperones in the initial stages, the histone chaperoning pathway may represent an efficient way to assemble a highly abundant cellular complex.

A gargantuan effort has been made regarding the discovery and characterisation of histone chaperoning proteins in the last 40 years since the discovery of Nucleoplasmin in 1978. This has culminated in the characterisation of a number of histone chaperones at atomic resolution in complex with their histone cargo, the spatio-temporal ordering of histone hand-off events (Figure 4A) and the realisation of the role of histone chaperones in development and disease. We expect that this will continue apace in the coming decade. The recent revolution in structure determination by cryo-EM, coupled to the biological importance of many large chaperoning complexes, such as CAF1, HIRA and FACT, means that it may not be long before we have a detailed understanding of how even the largest histone chaperoning complexes mediate interactions with their histone cargo. One key question still to be address is how histone hand-off between chaperoning components relates to the thermodynamic assembly of nucleosome in the context of the cellular environment (Figure 4B). Ongoing advances in live-cell imaging and super-resolution microscopy, combined with novel pulse-chase strategies and biochemical methods will hopefully result in new tools to probe the rapid kinetics of histone transfer between chaperones in living cells.

## Summary

- Core histones have a set of dedicated molecular chaperones that associate tightly with them from synthesis to chromatin incorporation.
- Under certain ionic buffering conditions, histones and DNA form nucleosomes *in vitro*. Histone chaperones, with their disparate domain structures, facilitate this process *in vivo*.
- Structural biology is spreading ever more light on the molecular details of histone–histone–chaperone interactions. To date this has been predominantly limited to individual domains, yet with the revolution in cryo-electron microscopy we anticipate greater understanding at the larger complex level in the near future.
- Transitions between chaperoning complexes are poorly understood, but fundamental to gaining an understanding of the thermodynamic rules governing nucleosome assembly.
- The fast kinetics of histone deposition makes it challenging to study in living cells, and thus new techniques are required to be able to test histone chaperoning models directly.

## Abbreviations

ARD: ankyrin repeat domain
CDAN1: Codanin-1
FACT: FACilitating Transcription
Nap1: nucleosome assembly protein 1
PCNA: proliferating cell nuclear antigen
PIP: PCNA interacting peptide
RPA: replication protein A
TLK1 and TLK2: Tousled-like kinases 1 and 2
UBN1: Ubinuclein-1
